# Unmet needs in recovery and rehabilitation: rethinking the limits of health care

**DOI:** 10.1192/j.eurpsy.2026.10294

**Published:** 2026-07-31

**Authors:** F. Oflaz

**Affiliations:** School of Nursing, Koç University, İstanbul, Türkiye

## Abstract

**Abstract:**

Despite progress in psychiatric treatment, *unmet needs* remain a persistent and structural problem in recovery and rehabilitation. These needs—such as social inclusion, autonomy, meaningful participation, housing, employment, and identity reconstruction—cannot be adequately addressed solely within health care systems. When recovery is framed primarily as a clinical responsibility, unmet needs risk being medicalised, individualised, and ultimately left unresolved.

This presentation adopts a critical, recovery-oriented perspective to argue that the dominance of health service–centred models actively contributes to the persistence of unmet needs. While mental health services are essential, many recovery needs emerge within social, political, and economic contexts that lie beyond the reach of clinical interventions. Institutional rehabilitation models may unintentionally reinforce dependency and marginalisation by overlooking structural determinants of recovery.

Drawing on trauma-informed and psychosocial frameworks, the presentation highlights the role of mental health nursing as both a clinical and advocacy practice. Addressing unmet needs requires cross-sectoral responsibility, community engagement, and a shift from service-led recovery to socially embedded, rights-based recovery approaches.

**Disclosure of Interest:**

None Declared

